# Evidence towards a continuum of impairment across neurodevelopmental disorders from basic ocular-motor tasks

**DOI:** 10.1038/s41598-022-19661-z

**Published:** 2022-10-03

**Authors:** Daniela Canu, Chara Ioannou, Katarina Müller, Berthold Martin, Christian Fleischhaker, Monica Biscaldi, André Beauducel, Nikolaos Smyrnis, Ludger Tebartz van Elst, Christoph Klein

**Affiliations:** 1 Department of Child and Adolescent Psychiatry, Psychotherapy, and Psychosomatics, Medical Centre - University of Freiburg, Faculty of Medicine, University of Freiburg, Freiburg, Germany; 2 Psychotherapeutisches Wohnheim für Junge Menschen Leppermühle, Buseck, Germany; 3 Institute of Psychology, University of Bonn, Bonn, Germany; 4 2nd Psychiatry Department, National and Kapodistrian University of Athens, Medical School, University General Hospital “ATTIKON”, Athens, Greece; 5 Laboratory of Cognitive Neuroscience and Sensorimotor Control, University Mental Health, Neurosciences and Precision Medicine Research Institute «COSTAS STEFANIS», Athens, Greece; 6 Department of Psychiatry and Psychotherapy, Medical Centre - University of Freiburg, Faculty of Medicine, University of Freiburg, Freiburg, Germany; 7 Department of Child and Adolescent Psychiatry, Medical Faculty, University of Cologne, Cologne, Germany

**Keywords:** Human behaviour, Psychiatric disorders

## Abstract

Findings of genetic overlap between Schizophrenia, Attention-Deficit/Hyperactivity Disorder (ADHD) and Autism Spectrum Disorder (ASD) contributed to a renewed conceptualization of these disorders as laying on a continuum based on aetiological, pathophysiological and neurodevelopmental features. Given that cognitive impairments are core to their pathophysiology, we compared patients with schizophrenia, ADHD, ASD, and controls on ocular-motor and manual-motor tasks, challenging crucial cognitive processes. Group comparisons revealed inhibition deficits common to all disorders, increased intra-subject variability in schizophrenia and, to a lesser extent, ADHD as well as slowed processing in schizophrenia. Patterns of deviancies from controls exhibited strong correlations, along with differences that posited schizophrenia as the most impaired group, followed by ASD and ADHD. While vector correlations point towards a common neurodevelopmental continuum of impairment, vector levels suggest differences in the severity of such impairment. These findings argue towards a dimensional approach to Neurodevelopmental Disorders’ pathophysiological mechanisms.

## Introduction

Schizophrenia is a complex chronic and disabling psychiatric disorder with neurodevelopmental origins^1^. Its usual emergence in adolescence or young adulthood has been explained by an interaction between distorted and normal brain developmental processes. Supportive evidence highlights prenatal and perinatal risk factors^2^ and schizophrenia-related genes early in neurodevelopment^3^. Furthermore, premorbid neurobehavioural deficits and structural, physiological, and neurochemical brain abnormalities may date back to childhood or earlier in high-risk samples^4^.

Despite neurodevelopmental contributions to its pathophysiology, schizophrenia has so far been considered as distinct aetiologically and nosologically from Neurodevelopmental Disorders (NDD), which define disturbances in the central nervous system development, with resulting abnormalities typically manifesting early in life. Among them, Autism Spectrum Disorder (ASD) denotes social interactions and communication impairments and restricted, repetitive patterns, that manifest from early childhood, whilst Attention-Deficit/Hyperactivity Disorder (ADHD) expresses core inattention, hyperactivity and impulsivity symptoms, identifiable from childhood^5^.

Despite their current nosological distinction, there is evidence of overlapping pathophysiological mechanisms between schizophrenia and NDD. Genomic studies isolated rare copy number variants common to schizophrenia, ADHD and ASD^6^ that could be associated with continuous variations in the neurodevelopmental impairment severity. Other key observations include overlapping environmental exposures affecting early brain development^7^, comorbidity, common motor, sensory and cognitive impairments^6^.

Such outcomes have supported the Neurodevelopmental Continuum Model of impairment^6^, according to which alterations in brain development may represent the overlapping underpinnings of different psychiatric disorders, such as schizophrenia, ADHD and ASD, which posit them along an aetiological and neurodevelopmental continuum. Within such continuum, NDD (including schizophrenia) occupy a gradient of decreasing neurodevelopmental impairment, resulting from age at onset, severity of associated cognitive impairment and persistence of functional impairment. While acknowledging that disorders differ along several other clinical dimensions Owen and O’Donovan^6^ hypothesised that within such continuum the degree (or gradient) of neurodevelopmental impairment is the most recognisable disorders’ feature.

Within this research framework, deficits in cognition have been identified across many psychiatric disorders, thus not being specific to any^8,9^.

However, the still fragmentary evidence presents cognitive deficits as secondary impairments in some psychiatric disorders. Cognitive impairments in depression and anxiety seem to depend on the current symptoms’ severity, the disorder course and the medication use^10,11^. If confirmed, these results would link cognitive constructs with the manifestations of the disorder but not with the risk of its development. However, opposite results have also been reported^10,11^ and no firm conclusion can be reached so far. Similarly, investigations in unaffected first-degree relatives provided inconclusive results on depression^12^, are absent on anxiety. Deficits in patients with obsessive compulsive disorder and their unaffected first-degree relatives are limited to cognitive inflexibility and motor impulsivity^13^. Conversely, results on planning, spatial working memory and volitional saccade performance are inconclusive^14,15^.

By contrast, cognitive deficits in schizophrenia, ADHD and ASD (and bipolar disorder) are enduring, not state-dependent, independent of the disorder subtype and the severity of the symptomatology^16–19^. They appeared in non-affected first-degree relatives^19–21^, linking the constructs with each disorder’s genetic liability. Given the relevance of cognitive deficits to these disorders and their pathophysiologies, their investigation could shed light on the disorders’ common (and unique) pathophysiologies*.*

Through eye movement recordings, many cognitive processes underlying saccade programming could be disentangled^22^. Subjects are instructed to make an eye movement to a peripheral visual stimulus in the prosaccade (PRO) paradigm, to its mirror location in the antisaccade (ANT) paradigm, towards a memorised stimulus in the memory-guided saccade (MEM) paradigm, or to refrain from making an eye movement in the fixation (FIX) paradigm. These tasks provide different operational definitions of the central constructs of processing speed, inhibition and intra-subject variability (ISV).

Starting with processing speed, prolonged antisaccade latency is systematically documented in schizophrenia, ADHD and ASD, consistent with a fronto-striatal pathophysiology, while typical PRO latency across clinical groups suggests mature saccade generation circuits^23–25^. Several studies also confirmed prolonged memory-saccade latency in SCZ compared to TD^23,25,26^ and ADHD^27^, results are less consistent for ADHD and ASD^25^.

Moving to inhibition, disproportionally frequent direction errors during ANT have been documented in schizophrenia, ADHD and ASD^23–25^, expressing disinhibition of a prepotent response. Similarly, frequent premature glances at memorised locations have been reported in schizophrenia or ADHD from separate^23,25,26^ or joint comparisons^26,27^ with controls, resulting from impaired interference control from disruption by competing events and responses. By contrast, findings have been inconsistent for ASD^22^.

Mixed results have been reported also for the FIX intrusive saccades rate^28,29^. Frequent intrusive saccades in schizophrenia^28^ could suggest difficulty in maintaining prolonged fixation in presence of distractors. Findings reported in a recent meta-analysis^13^ showed more intrusions during prolonged fixation without distractors in ADHD than controls, are conflicting from the condition with distractors. Results are no clearer regarding ASD^30,31^.

Ultimately, due to the repetitive presentation of analogous trials, saccade paradigms enable measurement of ISV. Corresponding to the short-term within-subject fluctuations in task performance, and, specifically, trial-to-trial variability of saccade latency, ISV can manifest through a high frequency of slow responses and fast anticipatory responses, expressing transient fluctuations and moment-to-moment variability of attention^32,33^. ISV has been attributed to neural noise, reduced myelination^34^ or periodicity of the nervous system^33^. One line of evidence linked increased ISV to fluctuating top-down attentional processes, following findings of increased ISV in patients with prefrontal lesions^35^. Another line of evidence linked increased ISV to catecholaminergic system dysregulations and consequent impairment in top-down control^36^. While being typically assessed through the standard deviation of response time (RT), a sensitive albeit non-specific measure of ISV, recent research emphasizes the need to more thoroughly investigate RT distributions to describe the nature of increased ISV of RT in psychiatric conditions. Indeed, increased ISV has been documented for schizophrenia^37,38^ and ADHD^31,33,39^ across saccade tasks and manual choice RT tasks, while results in ASD are less consistent^33,40–42^.

Finally, the Go/NoGo (GNG) task—requiring responding to target stimuli, not to non-target stimuli—is a useful comparison in virtue of its overlap in involved processes with saccade tasks, namely speed, ISV and response inhibition. Response selection and inhibition deficits have been found in schizophrenia and ADHD^43^. Results are ambiguous in ASD^44^ however, possibly due to the high comorbidity with ADHD that could not be diagnosed before DSM-5.

The present study investigated several cognitive trait markers in patients with schizophrenia, ADHD and ASD via basic ocular-motor tasks and a baseline manual response task, aiming to achieve the following goals: (1) to determine between-group similarities and differences on single ocular-motor and manual-motor measures, (2) to explore similarities and differences between clinical groups in their performance deviancy from healthy controls.

Based on above, the following hypotheses, contrasting scenarios and explorative research questions could be formulated.

I. Hypotheses on differences between patients and controls on single cognitive constructs:
  1. Impaired Inhibition in SCZ, ADHD and ASD.
  2. Increased Intra-subject variability (ISV) in SCZ and ADHD, unimpaired in ASD.
  3. Processing speed:
    3.1 Typical Processing speed in PRO in SCZ, ADHD and ASD.
    3.2 Decreased Processing speed in ANT, MEM and GNG in SCZ, ADHD and ASD.

To test the above hypotheses, we extracted the following variables: (1) Inhibition: direction errors in ANT, intrusive saccades in FIX, anticipatory saccades across PRO, ANT and MEM, commission errors in GNG; (2) ISV: Standard deviation of RT in PRO, AND, MEM and GNG, Ex-Gaussian model parameters σ and τ for PRO and GNG; (3) Processing Speed: Mean response time in PRO, ANT, MEM and GNG, Ex-Gaussian model parameter μ for PRO and GNG.

Noteworthy, while most of the hypotheses are supported by replicated findings, some studies failed to confirm atypical FIX inhibition towards distractors and prolonged MEM RT in ASD and ADHD. Similarly, results are unclear as to typical or prolonged manual RT during Go-stimuli in ASD.

II. Contrasting scenarios on differences in the profiles of deviancy from controls:
  1. Following Owen and O’Donovan prediction, ASD will show the overall most impaired and SCZ the least impaired performance.
  2. Based on the literature covering the specific tests we expected SCZ to show the most impaired and ASD the least impaired performance.
III. Explorative research questions about similarities between clinical groups in their profiles of deviancy:
  1. The three profiles of deviancy will be highly positively correlated, suggesting substantial commonalities in the quality of cognitive impairment between clinical groups.

While eye movements have been largely investigated in each of the three clinical groups in comparison to controls, and few studies comparing pairs of groups have also been published, regarding the simultaneous comparison of the three clinical groups on basic eye movement tasks the current study is to our knowledge so far unprecedented.

## Methods

### Participants

N = 103 participants were tested: 20 patients with schizophrenia (SCZ), from a residential home specialised in the treatment of adolescents with psychosis (Psychotherapeutisches Wohnheim für junge Menschen, Leppermühle); 28 with ADHD and 26 with ASD from the out-patient populations of the Department of Child and Adolescent Psychiatry, University Medical Centre Freiburg; 29 healthy controls (TD) from the departmental database, posted announcements and word of mouth (Table 1). Across groups, participants had normal or corrected-to-normal vision and no history of any neurologic condition. Both domains have been investigated during recruitment through a standardised set of questions. Furthermore, eye movement data were excluded from the final analyses if during the session any non-corrected visual problem became manifest.

**Table 1:** Group’s characteristics.

Variable	SCZ^a^	ADHD	ASD	TD	*F*_3,99_^b^	*p*^c^
N	20	28	26	29		
Age in years (mean ± SD)	19.8 ± 1.7	19.9 ± 1.4	19.7 ± 1.9	19.8 ± 1.6	0.094	0.979
Gender (% female)	29	46	4	59	7.834	**0.001**
RSPM (% correct)	47.2 ± 27.2	58.3 ± 25.8	72.2 ± 19.7	69.0 ± 16.4	5.920	**0.001**
Current medications (%)	100	28.6	0	–	–	–
CPE (mean ± SD)	451.8 ± 274.3	–	–	–		

*RSPM* Raven standard progressive matrices, *CPE* Chlorpromazine equivalent, *SD* standard deviation.

Column for each group reports Mean ± SD.

^a^Of the SCZ patients, one had an additional ADHD diagnosis (without taking psychostimulants), another one of autism with below-average IQ. Data of these two patients were included in the final analyses, since excluding them did not alter the results. Data from one patient from the SCZ group were excluded from analysis as he did not complete all tasks, which prevented the comparison of the performance between tasks. Patients from the ADHD group taking stimulant medication were medication-free for at least 24 h prior to testing, while patients from the SCZ group remained on their medications prior to and during testing.

^b^Analysis of variance (ANOVA) was used.

^c^Bold typeface = *p* < 0.05. *p* values for all variables indicate significance for differences between each clinical group and TD.

Patients with schizophrenia, ASD and ADHD had previously received a clinical diagnosis determined through expert clinical judgment. Patients from the SCZ group had a diagnosis of schizophrenia, schizophreniform or schizoaffective disorder. Patients from the ASD group had a diagnosis of childhood autism, atypical autism or Asperger Syndrome, following the administration of the Autism Diagnostic Observation Schedule (ADOS)^45^ and the Autism Diagnostic Interview-Revised (ADI-R)^46^. Patients from the ADHD group had a subtype diagnosis of ADHD (predominantly inattentive, hyperactive, combined, other or unspecified type), based on interviews with parents and children, behavioural observations and the Conner’s Rating scales^47^. Current symptomatology in ASD, ADHD and its absence in TD was assessed using the Social Responsiveness Scale (SRS)^48^ for ASD symptoms and the Conners’ Rating Scales for ADHD symptoms. Intelligence was measured across the four groups with two different instruments: using the Wechsler Intelligence Test (WISC-IV, WAIS-IV)^49^ in SCZ, and using the Grundintelligenztest Skala 2-Revision (CFT-20-R)^50^ in patients with ASD or ADHD as well as in the TD group. In addition, in order to guarantee an adequate between-group comparison in IQ, a 9-item version of the Raven Standard Progressive Matrices (RSPM)^51^, correlating with the original (long) version by r = 0.98 was taken by all participants^52^. Participants’ written informed consent was obtained, in addition to parent consent for minors. The study was given ethical approval by the University of Freiburg Ethics Committee (ethical approval: No. 124/17).

### Procedures

Participants were administered a battery of eye movement tasks in counterbalanced order within each group. A dominant eye test determined ocular preference, the Edinburgh Handedness Inventory^53^ assessed participant’s handedness.

Participants sat inside a lit cabin, in front of a flat, 24 in. LCD screen monitor with a resolution of 1920 × 1080 pixels and a refresh rate of 60 Hz, at a viewing distance of 90 cm, with stabilised head via a forehead and chin rest. The cabin’s illumination level was maintained between 70 and 80 lx. Each task started with standardised instructions by one examiner sitting next to the participant, followed by practice trials. Administration of experimental trials followed, while the experimenter moved laterally behind the participant, to ascertain participant’s stillness and appropriate task execution. A second examiner sitting in an adjacent room, monitored eye movements to ensure adequate gaze recording and participant’s alertness.

### Tasks

While a review of the complex cognitive processes underlying task performance is beyond our scope, we would nevertheless like to highlight some cognitive aspects of the employed tasks.

The performance during ANT is believed to reflect inhibition of an automatic response, sensorimotor transformation mechanisms to convert the stimulus spatial location to the opposite visual field and control mechanisms to facilitate the generation of a volitional saccade to the newly calculated location.

In PRO, there is a direct correspondence between sensory stimulation (stimulus) and motor response (to look at the stimulus). This results in a considerably faster programming of saccades. Thus, the additional time to initiate a volitional in comparison to a visually-guided saccade represents the extra time for the sensorimotor transformation or volitional decision-making processes^36^.

Furthermore, including offsetting the central stimulus some 200 ms before presenting the peripheral stimuli allows investigating the mechanisms behind reduction in the saccade latency, which have been hypothesised to reflect release of fixation and motor preparation processes, that occur between the offset of the central fixation and the onset of the peripheral stimulus and a fixation offset effect (FOE) specific to the ocular-motor system^54^. Its application to both PRO and ANT allows to test the generalisation of such processes across different task difficulty levels.

MEM requires the suppression of a glance toward the presentation of the peripheral stimulus. It also activates the processes of maintenance of the visuospatial location of the stimulus and the generation of a volitional saccade to the now unmarked location in the visual field^55^.

Furthermore, FIX activates inhibition skills while not engaging sensorimotor transformation mechanisms for the generation of a saccade^56^.

Ultimately, the GNG task involves rapid response preparation following a Go-stimulus appearance, inhibition of an automatic manual response towards a NoGo-stimulus and conflict monitoring operations when response tendencies interfere with each other.

All tasks were programmed using Experiment Builder (SR Research Ltd., version 2.1.140). A representation and a description of the tasks are provided in Supplementary Figs. S1–S4.

### Recording of eye movements

Eye movements were recorded binocularly with the EyeLink 1000 Plus system (SR Research, Mississauga, ON, Canada), at a sampling rate of 1000 Hz and spatial resolution of 0.01°. A nine-point manual calibration method was used to calibrate and validate the eyes’ position. Calibration was accepted with gaze inaccuracies ≤ 1° binocularly. Additionally, trials in each task were interleaved by a drift correction using a central fixation point, accepted if the gaze accuracy was ≤ 0.5° binocularly. Recordings were recalibrated whenever necessary.

### Primary data processing

Eye movement recordings were analysed offline. Analyses were performed for one eye, depending on subject ocular preference and the eye that, separately for each task, produced the most stable and noise-free signal. The eye trace quality was established via comparison of the two eyes on calibration, validation and drift corrections. This was combined with information derived from the test of ocular preference and a final decision was made.

Saccades and fixations were automatically detected according to a velocity-based method (EyeLink algorithm). Saccade onset was defined as peak velocity > 25°/s and amplitude > 1°, fixations as any period that was not a blink or a saccade. The EyeLink Host PC Software marks blink events by flagging the onset and offset of periods when the pupil is missing from the camera image, or is too small for its center to be reliably computed. The pupil size is determined by the number of pixels on the camera sensor that are below the pupil threshold (a grayscale value set by one experimenter during participant setup).

Across saccade tasks, trials in which blinks occurred between onset and offset of the first correct saccade were excluded from analysis.

### Dependent variables

Based on the previously listed (i) Stated hypotheses on differences between patients and controls on single cognitive constructs.

#### Inhibition

Across PRO, ANT and MEM, we extracted the direction errors, if the first saccade after appearance of the peripheral stimulus (PRO, ANT) or the disappearance of the fixation cross (MEM) was in the wrong direction (away from the target in PRO; toward the target in ANT; away from the memorised target location in MEM).

In PRO and ANT, we defined anticipatory saccades as saccades with onset after 80 ms and before 300 ms from the target onset and express saccades as saccades with onset between 81 and 130 ms. In MEM, saccades were coded as anticipatory if executed in the direction of the peripheral target during the memory phase, as correct if they followed the disappearance of the central cross (cue) and were directed towards the location of the remembered cue.

In FIX, intrusive saccade errors were defined as unnecessary rapid shifts in horizontal eye position that exceeded 1° in amplitude, away from the target for the block without distractors or in the distractor’s direction for the blocks with distractors.

In the GNG task error measures included percentage of omission errors (missed responses) in Go-trials and of commission errors (false alarms) in No-Go trials.

Across tasks, error rate was quantified as the number of error trials divided by the total number of usable trials, then multiplied by 100.

#### Intra-subject variability (ISV)

ISV was defined as the standard deviation (SD) of response times of correct saccades (PRO, ANT, MEM) and correct manual responses (GNG).

To analyse ISV further, the ex-Gaussian analysis was performed. In fact, while the RT distribution shows an asymmetrical positive skew that refutes the Gaussian assumption of normality^57^, the ex-Gaussian distribution model allows to decompose the RT distribution into Gaussian and exponential components, providing a better fit to the actual RT spread^58^. Noteworthy, although their psychological interpretation has been questioned^59^ the ex-Gaussian σ and τ have been shown to reflect different underlying processes, namely sensory-motor and perceptual attentional processing (for σ) or fluctuation resulting from poor suppression of the default mode network (for τ)^33^.

Coherently, the parameters sigma (σ) and tau (τ), signifying the SD of the Gaussian and of the exponential component, respectively, were extracted from PRO and GNG RTs, given the relatively high frequency of correct trials in both tasks.

#### Processing speed

Processing speed was defined as mean response times of correct responses across different tasks. Saccadic response time was defined as the time between target onset (PRO, ANT) or central fixation offset (MEM) and correct saccade onset. Manual response time for Go-trials (correct responses) from the GNG task was measured as the time between onset of Go-stimuli and button press. Furthermore, the ex-Gaussian parameter mu (μ), reflecting the mean of the Gaussian component of the RT distribution, was extracted from PRO and GNG RTs of correct responses.

### Statistical analyses

A priori hypotheses were tested via repeated measures ANOVA, with the dependent variables testing different facets of each construct as the levels of the within-subject factor named after the construct. For ISV the repeated measures ANOVA were performed twice: (1) SDRT across the four tasks and (2) SDRT for ANT and MEM separately from σ and τ for PRO and GNG. Similarly, for processing speed the analyses were repeated, including (1) mean RT across the four tasks and (2) mean RT for ANT and MEM and μ for PRO and GNG. In the case of violations of the sphericity assumptions Greenhouse–Geisser corrections of degrees of freedom were applied and the corresponding test statistics (F, *p*) reported. Following significant GROUP main effects across all dependent variables, differences between each clinical group and healthy controls for single dependent variables were tested via planned contrast for single dependent variables, adopting a significance level of α = 0.05 (two-tailed). For the remaining multivariate analyses, referring to control variables and exploration, we adopted a significance level of α = 0.01 (two-tailed) in order to reduce the probability of false positive results.

Furthermore, repeated-measures ANOVAs were run as a full factorial design, comprising the between-subject factor “GROUP” (TD, SCZ, ADHD, ASD) and five within-subject factors: “THREETASK” (PRO, ANT, MEM), to test saccade task main effect, GAPOVERLAP (gap, overlap) to test the gap effect during PRO, ANT and FIX, its (in)dependency of saccade task (TWOTASK (PRO, ANT)), TARGETLOCATION (PRO, ANT: ± 7°) and TARGETECCENTRICITY (MEM: ± 2.5°, ± 5°, ± 7.5°, ± 10°, ± 12.5°, ± 15°; FIX: ± 4, ± 7°), to explore the main effect of target and distractor location, its interaction with GROUP, task and GAPOVERLAP, and ultimately BLOCK (FIX: without distractors, with distractors) to explore the main effect of FIX condition.

Task parameters were grouped in two categories, being continuous and categorical variables. Continuous variables included all dependent variables (mean, standard deviation and ex-Gaussian parameters of RT, percentage and frequency values). Categorical variables included the between-subject factor GROUP and all within-subject factors (THREETASK, GAPOVERLAP, TARGETECCENTRICITY, BLOCK).

Across variables the normality assumption was evaluated using the Levene’s test. Parametric comparisons were accomplished even following the few cases of violation of the normality assumption, as frequently reported in the literature in light of its known limited and manageable risks and the robustness of parametric tests against normality violation^60^.

In the context of the Ex-Gaussian analyses and in order to exclude potential ISV confounders, RT of manual correct responses to Go-trials (GNG) and regular correct saccades (PRO) were “residualised” before extracting σ and τ. To do so, we ran a multivariate analysis, including “trial index” as covariate, to control for potential confounders of Type I ISV (fluctuations in performance arising purely from within an organism when presented with the same stimulus in the same situation at two points in time) effects of linear trends, and GAPOVERLAP and TARGETLOCATION from PRO as factors to control for Type III ISV effect of variations in task conditions. This residualisation procedure thus removed all components of ISV due to task, stimulus manipulations or trends^32^.

Given the between-group differences on gender distributions and between SCZ and any other group on the IQ score and medication status, all variables were included as covariates after mean-centring^61^, repeating analyses by means of ANCOVAs. In the inclusion of IQ as covariate we sticked with a common statistical recommendation, which has also been discussed controversially^62^.

Potential antipsychotic medication effects on the performance of SCZ were further tested via correlation analyses between Chlorpromazine equivalent (CPE) and dependent variables.

Differences between each clinical group and TD were calculated for each dependent variable using independent t-tests, thus obtaining three vectors of group differences (SCZ-vs-TD; ASD-vs-TD; ADHD-vs-TD) as expressed through t-values. This procedure was accomplished for all measures of SD after controlling statistically for the corresponding means by residualisation. These difference vectors were processed further in two ways and with two complementary aims. *First*, *vector levels* were defined as the average of the t-values constituting a vector and were compared between clinical groups using paired-sample t-tests to quantify the *relative degree of impairment* of each clinical group. *Second*, *vector correlations* were obtained by correlating the profiles of deviancy (t-test-based vectors) between clinical groups (SCZ–ADHD, SCZ–ASD, ADHD–ASD) using Pearson’s r to quantify the qualitative *similarity of impairment* across clinical groups.

In order to examine the potential dependencies of levels or similarities of impairments on the inclusion of IQ, vector correlations/levels were re-calculated following partialling all dependent variables for the RSPM scores. Vector correlations/levels were, furthermore, re-calculated with RSPM scores included as an additional variable. Ex-Gaussian parameters were derived using the retimes package for the statistical package RStudio (version 1.2.1335). All statistical analyses were performed with SPSS software, Version 24 (SPSS Institute Inc., Cary, NC, USA). All methods were carried out in accordance with relevant guidelines and regulations.

## Results

Descriptive statistics for all dependent measures are presented in Table 2.

**Table 2:** Descriptive statistics.

Task	Dependent variable	SCZ	ADHD	ASD	TD
		Mean ± SD	Mean ± SD	Mean ± SD	Mean ± SD
GNG	Mean RT	478 ± 79	436 ± 52	452 ± 86	431 ± 33
	SDRT	120 ± 50	98 ± 41	94 ± 39	81 ± 20
	Mu	363 ± 93	342 ± 46	366 ± 70	360 ± 33
	Sigma	61 ± 54	43 ± 35	45 ± 23	45 ± 16
	Tau	115 ± 70	95 ± 55	86 ± 41	71 ± 25
	Commission errors	32 ± 15	27 ± 16	25 ± 14	18 ± 13
	Omission errors	6 ± 11	1 ± 3	3 ± 9	1 ± 2
PRO	CS	87 ± 10	93 ± 5	92 ± 9	94 ± 5
	Mean RT	247 ± 38	241 ± 38	232 ± 29	243 ± 29
	SDRT	89 ± 22	78 ± 30	69 ± 26	71 ± 20
	Mu	160 ± 41	170 ± 34	176 ± 32	183 ± 24
	Sigma	43 ± 21	35 ± 17	40 ± 16	39 ± 13
	Tau	84 ± 27	71 ± 34	56 ± 27	60 ± 25
	AS	7 ± 8	4 ± 4	5 ± 6	3 ± 3
ANT	CS	53 ± 23.6	72 ± 16.9	77 ± 16.5	84 ± 10.5
	Mean RT	410 ± 68	373 ± 67	362 ± 68	356 ± 43
	SDRT	113 ± 26	102 ± 29	97 ± 34	92 ± 24
	AS	7 ± 8	3 ± 4	2 ± 3	2 ± 3
	DE	32 ± 19	23 ± 15	19 ± 14	11 ± 9
MEM	CS	17 ± 24	61 ± 21	66 ± 23	75 ± 13
	Mean RT	450 ± 103	427 ± 94	413 ± 84	387 ± 55
	SDRT	195 ± 85	186 ± 85	166 ± 106	147 ± 58
	AS	49 ± 18	32 ± 17	31 ± 22	20 ± 13
	AS_CS	40 ± 15	28 ± 17	26 ± 19	18 ± 11
FIX	Intr. Saccade NO-D	1 ± 1	1 ± 1	1 ± 3	1 ± 1
	Intr. Saccade D	4 ± 4	2 ± 3	3 ± 4	1 ± 2

*Mean RT* mean response time, *SDRT* standard deviation of response time, *CS* correct responses, *AS* anticipatory saccades, *DE* direction errors from the ANT task, *AS_CS* correct anticipatory saccades from the MEM task, *Intr. Saccade NO-D* intrusive saccades from block without distractors of the FIX task, *Intr. Saccade D* intrusive saccades from block with distractors of the FIX task.

Column for each group reports Mean ± SD (standard deviation).

### General accuracy

A significant between-group difference in the proportion of correct saccades was found across tasks. Compared to TD, SCZ made less correct responses in all tasks, ADHD in ANT and MEM, never ASD (see Table 4).

### Inhibition

The repeated measures ANOVAs for the different measures of inhibition highlights a significant GROUP main effect (*F*_3,99_ = 16.019, *p* < 0.0001, *η*_p_^2^ = 0.327, see Table 3).

**Table 3:** Significant within-subject factors and group main effects.

	Dependent variable	Within-Subject Factor Main effect			GROUP Main effect
		F	p^a^	η_p_^2^	
**Within-subject factor (construct)**					
Inhibition	Errors across tasks	F_3.00,296.71_ = 182.8	** < 0.0001**	0.649	Patients > TD
ISV	SDRT across tasks	F_1.64,162.04_ = 87.1	** < 0.0001**	0.468	Patients > TD
ISV	SDRT—Sigma, Tau	F_2.30,227.78_ = 311.5	** < 0.0001**	0.759	Patients > TD
Processing speed	Mean RT across tasks	F_2.62,258.94_ = 310.4	** < 0.0001**	0.759	Patients > TD
Processing speed	Mean RT—Mu	F_2.62,259.33_ = 397.4	** < 0.0001**	0.801	n.s.
**Within-subject factor (task)**					
Threetask (PRO, ANT, MEM)	Mean RT	F_1.83,181.29_ = 319.8	** < 0.0001**	0.764	Patients > TD
	SDRT	F_1.55,153.29_ = 77.6	** < 0.0001**	0.439	n.s.
	AS	F_1.08,107.31_ = 340.0	** < 0.0001**	0.775	Patients > TD
Gapoverlap (PRO, ANT)	Mean RT	F_3,99_ = 511.8	** < 0.0001**	0.839	n.s.
	SDRT	F_3,99_ = 144.0	** < 0.0001**	0.595	n.s.
Gapoverlap (ANT)	DE	F_3,99_ = 38.0	** < 0.0001**	0.280	n.s.
Targeteccentricity (MEM)	Mean RT	F_4.27,364.00_ = 7.4	** < 0.0001**	0.080	Patients > TD at smaller eccentricities
	SDRT	F_4.33,315.96_ = 4.0	**0.003**	0.052	n.s.
	AS	F_3.75,367.77_ = 9.3	** < 0.0001**	0.086	Patients > TD across eccentricities
Block (FIX)	Intr. Saccade	F_3,99_ = 47.5	** < 0.0001**	0.324	Patients > TD in block with distractors
Gapoverlap (FIX)	Intr. Saccade	F_3,99_ = 21.3	** < 0.0001**	0.177	n.s.
Targeteccentricity (FIX)	Intr. Saccade	F_3,99_ = 4.6	**0.035**	0.044	n.s.

*ISV* Intra-subject variability*, Mean RT* Mean response time*, SDRT* Standard deviation of response time*, Errors across tasks* anticipatory saccade in PRO, anticipatory saccade and direction error in ANT, anticipatory saccade in MEM, intrusive saccade in FIX and commission error in GNG, *SDRT across tasks* SDRT across tasks standard deviation of response time across PRO, ANT, MEM and GNG, *SDRT—Sigma, Tau* standard deviation of response time from ANT and MEM and Sigma and Tau from PRO and GNG, *Mean RT across tasks* Mean response time across PRO, ANT, MEM and GNG, *Mean RT—Mu* Mean response time from ANT and MEM and Mu from PRO and GNG, *AS* anticipatory saccade*, DE* direction error from ANT, *Intr. Saccade* Intrusive Saccade from FIX, *TD* healthy controls, *n.s.* not significant*.* F values are based on Greenhouse–Geisser and its correction of degrees of freedom.

^a^*Bold typeface* = *p* < 0.05. p values for all variables indicate significance for differences between within-subject factor conditions.

The highest proportion of direction errors was found in ANT, of anticipatory saccades in MEM, more in patients than controls (direction errors: *F*_3.07,101.15_ = 8.967, *p* < 0.0001, *η*_p_^2^ = 0.214; anticipatory saccades: *F*_3.25,107.31_ = 9.519, *p* < 0.0001, *η*_p_^2^ = 0.224, see Table 3).

Compared to TD, patients with SCZ, ADHD and ASD showed more direction errors during ANT and anticipatory saccades during MEM. Considering only the MEM anticipations towards the target direction, differences between ASD and TD became marginally significant (*p* = 0.056, *d* = 0.5, see Table 4).

**Table 4:** Planned contrasts between TD and clinical groups, Post-Hoc comparisons between clinical groups.

Task	Dependent variable	SCZ-TD			ADHD-TD			ASD-TD			Post-Hoc comparisons
		*t*_*99*_^a^	*p*^b^	*d*^c^	*t*_*99*_^a^	*p*^b^	*d*^c^	*t*_*99*_^a^	*p*^b^	*d*^c^	
GNG	Mean RT	− 2.513	**0.014**	0.834	− 0.338	0.736	0.133	− 1.239	0.218	0.337	n.s.
	SDRT	− 3.598	**0.001**	1.123	− 1.652	0.102	0.513	− 1.232	0.221	0.414	n.s.
	Mu	− 0.168	0.867	0.047	1.106	0.272	0.450	− 0.421	0.675	0.129	n.s.
	Sigma	− 1.704	0.091	0.450	0.139	0.889	0.045	0.035	0.972	0.016	n.s.
	Tau	− 3.114	**0.002**	0.907	− 1.851	0.067	0.565	− 1.131	0.261	0.444	n.s.
	Commissions errors	− 3.133	**0.002**	0.939	− 2.276	**0.025**	0.600	− 1.679	0.096	0.488	n.s.
	Omission errors	− 2.526	**0.013**	0.712	− 0.218	0.828	0.163	− 1.250	0.214	0.363	n.s.
PRO	CS	3.405	**0.001**	0.981	0.757	0.451	0.289	1.139	0.258	0.32	SCZ > ADHD
	Mean RT	− 0.436	0.664	0.129	0.208	0.836	0.055	1.210	0.229	0.377	n.s.
	SDRT	− 2.472	**0.015**	0.849	− 1.030	0.305	0.269	0.359	0.721	0.105	SCZ > ASD
	Mu	2.447	**0.016**	0.726	1.470	0.145	0.431	0.838	0.404	0.263	n.s.
	Sigma	− 2.406	**0.018**	0.631	− 0.657	0.513	0.186	− 1.553	0.124	0.474	n.s.
	Tau	− 3.069	**0.003**	0.917	− 1.574	0.119	0.398	0.338	0.736	0.099	SCZ > ASD
	AS	− 3.091	**0.003**	0.858	− 0.735	0.464	0.305	− 1.655	0.101	0.49	n.s.
ANT	CS	6.317	**<0.0001**	1.816	2.807	**0.006**	0.896	1.581	0.117	0.527	SCZ > ADHD, ASD
	Mean RT	− 3.057	**0.003**	1.002	− 1.052	0.295	0.307	− 0.378	0.706	0.112	SCZ > ASD
	SDRT	− 2.586	**0.011**	0.86	− 1.319	0.190	0.379	− 0.695	0.489	0.184	n.s.
	AS	− 4.259	**<0.0001**	1.044	− 1.097	0.275	0.357	− 0.295	0.768	0.126	SCZ > ADHD, ASD
	DE	− 5.126	**<0.0001**	1.498	− 3.020	**0.003**	0.932	− 2.193	**0.031**	0.722	SCZ > ASD
MEM	CS	9.803	**<0.0001**	3.101	2.651	**0.009**	0.824	1.663	0.099	0.496	SCZ > ADHD, ASD
	Mean RT	− 2.591	**0.011**	0.808	− 1.752	0.083	0.512	− 1.135	0.259	0.368	n.s.
	SDRT	− 2.000	**0.048**	0.671	− 1.729	0.087	0.538	− 0.867	0.388	0.234	n.s.
	AS	− 5.757	**<0.0001**	1.939	− 2.568	**0.012**	0.797	− 2.362	**0.020**	0.641	SCZ > ADHD, ASD
	AS_CS	− 4.929	**<0.0001**	1.776	− 2.559	**0.012**	0.767	− 1.937	0.056	0.535	SCZ > ASD
FIX	Intr. Saccade NO-D	0.94	0.783	0.44	0.326	0.988	0.07	1.365	0.524	0.288	n.s.
	Intr. Saccade D	2.405	0.873	0.698	1.392	0.507	0.329	1.065	0.711	0.261	n.s.

*Mean RT* mean response time, *SDRT* standard deviation of response time, *CS* correct responses, *AS* anticipatory saccades, *DE* direction errors, *AS_CS* correct anticipatory saccades, *Intr. Saccade NO-D* intrusive saccades from block without distractors of the FIX task, *Intr. Saccade D* intrusive saccades from block with distractors of the FIX task, *n.s.* not significant.

^a^t-test for planned contrasts was used.

^b^Bold typeface = *p* < 0.05. *p* values for all variables indicate significance for differences between each clinical group and TD. ^c^Cohen’s *d* was used to calculate the effect size.

The proportion of ANT direction errors and MEM anticipatory saccades towards the target direction was descriptively higher in SCZ than ASD. When including all MEM anticipations (independently of target direction) SCZ showed more anticipations than ADHD and ASD (see Table4 and Supplementary Table S1). Furthermore, SCZ only also showed more anticipations than TD during PRO and ANT (see Table 4).

During FIX, the block with distractors elicited more intrusive saccades than the one without, differently in the four groups (BLOCK × GROUP, *F*_3,99_ = 3.797, *p* = 0.013, *η*_p_^2^ = 0.103, see Table 3).

SCZ and ASD made more intrusive saccades than TD in the distractor direction, while patients did not differ from controls in the block without distractors (see Table 3).

The proportion of GNG commission errors was significantly higher in SCZ and ADHD than TD. SCZ only additionally presented more omissions than TD (see Table 4 and Supplementary Table S1).

**Figure 1:**
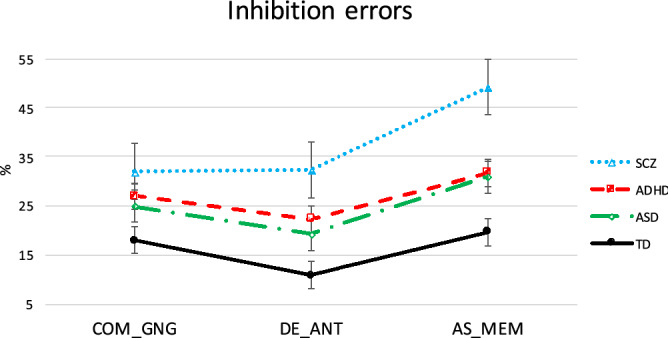
The graph represents the percentage of inhibition errors across tasks on which clinical groups most deviated from healthy controls, namely commission errors from the GNG task (“COM_GNG”), anticipatory saccades from the MEM task (“AS_MEM”) and regular direction errors from the ANT task (“DE_ANT”). The error bars represent one standard error of the mean. The effect sizes from the between-group subject ANOVA are also reported here. COM_GNG: *η*_p_^2^ = 0.098, DE_ANT: *η*_p_^2^ = 0.139, AS_MEM: *η*_p_^2^ = 0.148.

The results from the between-group comparisons on the percentage of ANT direction errors, MEM anticipations and GNG commission errors are shown in Fig. 1.

When controlling for gender, IQ or CPE, results did not vary much, except differences between SCZ and TD on SDRT during MEM that turned out somewhat less significant when controlling for IQ (*t*_99_ = 1.997, *p* = 0.049). Correlation coefficients between dependent variables and CPE were all statistically non-significant, and associated with a small to moderate effect size (see Supplementary Table S2).

In sum, the hypothesised inhibition deficit was found for SCZ, ADHD and ASD using the proportion of ANT direction errors and MEM anticipations; it was found for SCZ and ASD also using the frequency of FIX intrusive saccades (towards distractors) and for SCZ and ADHD also using the proportion of GNG commission errors.

### Intra-subject variability (ISV)

Repeated measures ANOVA including across-tasks SDRT (*F*_3,99_ = 4.701, *p* = 0.004, *η*_p_^2^ = 0.125) and ex-Gaussian parameters (*F*_3,99_ = 4.039, *p* = 0.009, *η*_p_^2^ = 0.109) lead to significant results (see Table 3).

Across groups, the largest SDRT was found in MEM, the smallest in PRO (see Table 3). Increased SDRT across saccade tasks was found in SCZ compared to TD, descriptively also compared to ASD during PRO (see Table 4 and Supplementary Table S1). After excluding the one SCZ patient with comorbid ASD and below-average IQ, differences between SCZ and TD on PRO shrank to a trend with a medium effect size (*t*_98_ = − 1.932, *p* = 0.062, *d* = 0.596).

In ADHD, SDRT for ANT and MEM lay in between that of SCZ, ASD and TD, not significantly differing from either group. ASD never differed from TD.

Likewise, in the GNG task SCZ presented larger SDRT than TD, while ADHD and ASD did not differ from either group (see Table 4 and Supplementary Table S1).

**Figure 2:**
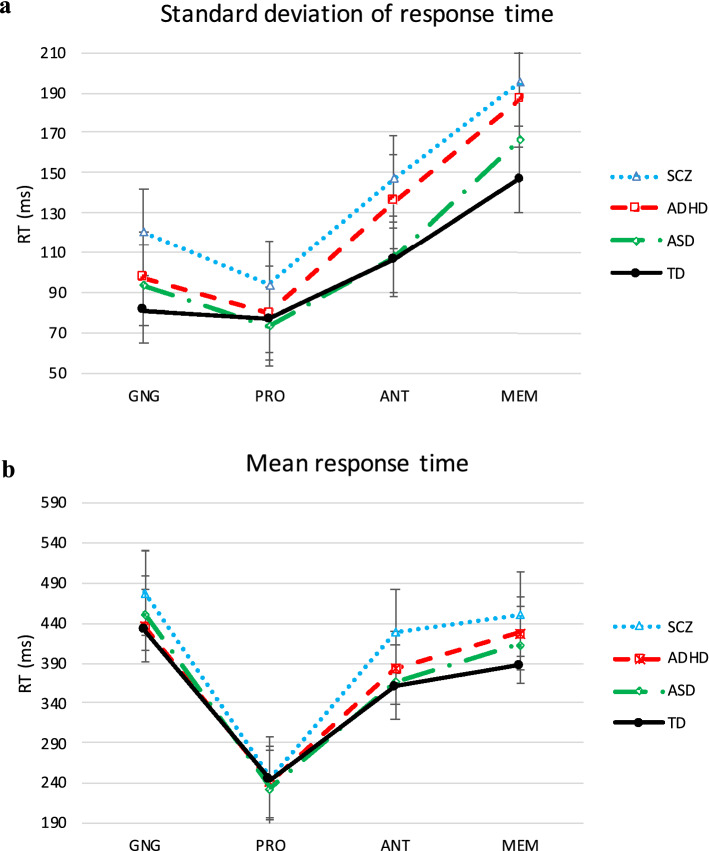
(**a**) Standard deviation and (**b**) Mean of RT for correct response from GNG, PRO, ANT, MEM. Error bars represent one standard error of the mean. The effect sizes from the between-group subject ANOVA are also reported here. Standard deviation of RT: GNG: *η*_p_^2^ = 0.117, PRO: *η*_p_^2^ = 0.041, ANT: *η*_p_^2^ = 0.103, MEM: *η*_p_^2^ = 0.04. Mean RT: GNG: *η*_p_^2^ = 0.069, PRO: *η*_p_^2^ = 0.026; ANT: *η*_p_^2^ = 0.096; MEM: *η*_p_^2^ = 0.068.

The results from the between-group comparisons on SDRT across tasks are shown in Fig. 2a.

Smaller μ together with larger σ and τ were found in SCZ compared to TD. While ADHD and ASD did not differ from either group, the former showed non-significantly larger τ than TD, a difference associated with medium effect size (see Table 4).

**Figure 3:**
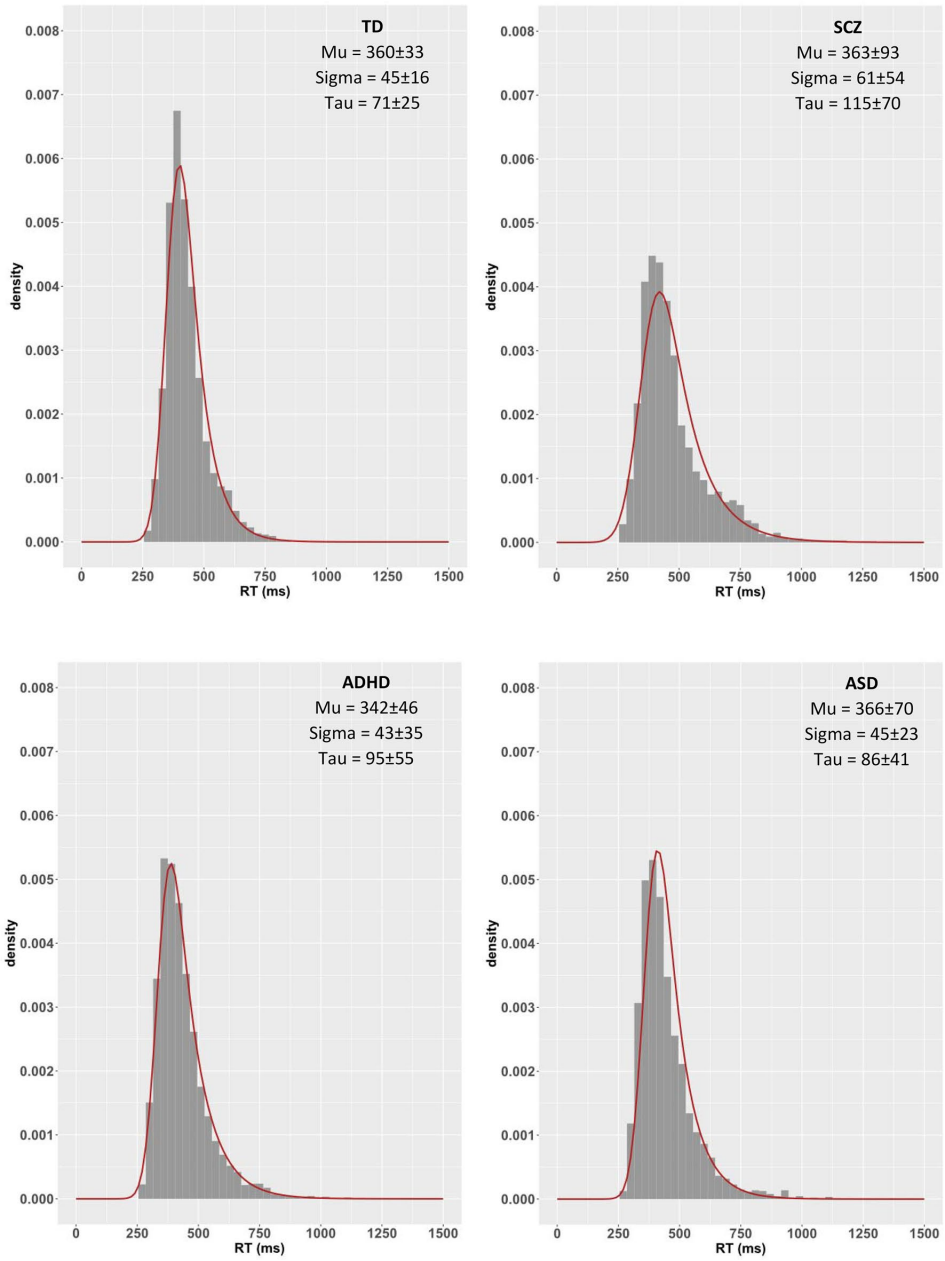
The four plots show the RT distributions in the four groups in GNG. The RT are plotted in the x-axis and the density in the y-axis. The ex-Gaussian model fit on the RT distribution is also plotted (red line), and is calculated based on the average of each ex-Gaussian parameter from all participants, separately for the three parameters and the four groups. Graphs were created using the *ggplot2* package for the open-source statistical package RStudio. The effect sizes from the between-group subject ANOVA are also reported here. Mu: *η*_p_^2^ = 0.026, Sigma: *η*_p_^2^ = 0.041, Tau: *η*_p_^2^ = 0.093.

In the GNG task, SCZ presented larger σ and τ than TD, ADHD had marginally larger τ than TD but still the second largest τ, while ASD did not differ from TD, ADHD or SCZ (see Tables 3 and 4). RT distributions and ex-Gaussian fits are plotted in Fig. 3.

In sum, the hypothesised increased ISV was found for SCZ using SDRT across tasks and the ex-Gaussian parameters σ and τ across PRO and GNG. It was never significantly increased in ADHD and ASD. For ADHD differences from TD were still associated with a moderate effect size using τ in GNG and the SDRT in MEM.

### Processing speed

When including mean RT across tasks, the GROUP main effect was significant (*F*_3,99_ = 3.734, *p* = 0.014, *η*_p_^2^ = 0.102). Conversely, results were not significant (*F*_3,99_ = 1,473, *p* = 0.226, *η*_p_^2^ = 0.043) for the ex-Gaussian parameter μ for PRO and GNG.

ANT and MEM required longer RT than PRO, differently so in the four groups (THREETASK × GROUP *F*_5.49,181.29_ = 2.272, *p* = 0.044, *η*_p_^2^ = 0.064, see Table 3).

Typical average prosaccade RT was found in all groups. Conversely, SCZ showed prolonged antisaccade and memory-saccade RT than TD and descriptively prolonged antisaccade RT than ASD. Conversely, ADHD and ASD did not differ from TD on ANT or MEM average RT (see Table 4 and Supplementary Table S1).

Finally, patients with SCZ were slower manual responders than TD, while ADHD and ASD did not differ from either group.

The results from the between-group comparisons on SDRT across tasks are shown in Fig. 2b.

In sum, the hypothesised decreased processing speed was found only for SCZ using mean RT across ANT, MEM and GNG.

### Additional results

The average RT decreased in gap compared to overlap trials, more in PRO than ANT (TWOTASK × GAPOVERLAP, *F*_3,99_ = 29.129, *p* < 0.0001, *η*_p_^2^ = 0.229), across all groups alike. Overlap trials also elicited larger SDRT than gap trials, more in ANT than PRO (TWOTASK × GAPOVERLAP: *F*_3,99_ = 8.420, *p* = 0.005, *η*_p_^2^ = 0.079), similarly across groups (see Table 3). Noteworthy, the GAPOVERLAP main effect on SDRT disappeared when controlling for mean RT in PRO (*F*_3,99_ = 1.168, *p* = 0.282, *η*_p_^2^ = 0.012) and ANT (*F*_3,99_ = 1.426, *p* = 0.235, *η*_p_^2^ = 0.014). By contrast, the gap effect on mean RT shrank (PRO: *F*_3,99_ = 3.073, *p* = 0.083, *η*_p_^2^ = 0.030) or remained significant (ANT: *F*_3,99_ = 10.076, *p* = 0.002, *η*_p_^2^ = 0.094) when co-varying for SDRT.

The proportion of ANT direction errors and of PRO and ANT anticipations was higher in gap than overlap trials, similarly across groups. Furthermore, in FIX intrusive saccades towards distractor increased in gap as compared to overlap trials across all groups alike (see Table 3).

In MEM the highest proportion of anticipatory saccades occurred with targets at 2.5° and 10°, but differently so between groups (*F*_11.26,367.77_ = 1.883, *p* = 0.039, *η*_p_^2^ = 0.054, see Table 3 and Supplementary Fig. S5). TD showed frequent anticipations when the target appeared at 2.5° only, while clinical groups presented a more homogeneous pattern across all six eccentricities.

On the mean RT, greater differences between all clinical groups and TD were manifest at smaller target eccentricities (*F*_12.82,363.10_ = 2.372, *p* = 0.005, *η*_p_^2^ = 0.077, see Table 3 and Supplementary Fig. S6).

### Vector levels and vector correlations

**Figure 4:**
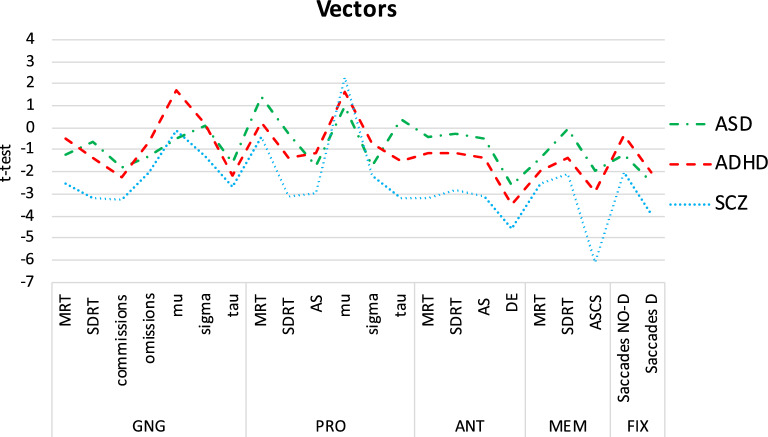
Vectors of deviancy from controls based on multiple comparisons of each clinical group versus TD on all dependent variables. *SD standard deviation, *AS anticipatory saccades, *Saccades No-D FIX block without distractors, *Saccades D FIX block with distractors.

Vector levels confirmed the highest degree of impairment in SCZ, compared to ASD (*t*_21_ = − 6.260, *p* < 0.0001, *d* = 1.335, *η*^2^ = 0.308) and even more so to ADHD (*t*_21_ = -8.766, *p* < 0.0001, *d* = 1.869, *η*^2^ = 0.466), while ASD and ADHD (*t*_21_ = − 1.060, *p* = 0.301, *d* = 0.226, *η*^2^ = 0.013) did not differ significantly. Despite level differences, the three profiles of deviancy from controls were highly positively correlated between the clinical groups. This held consistently for SCZ and ADHD (*r*_22_ = 0.896, *p* < 0.0001, *d* = 4.036, *η*^2^ = 0.802), SCZ and ASD (*r*_22_ = 0.660, *p* = 0.001, *d* = 1.757, *η*^2^ = 0.436) and ADHD and ASD (*r*_22_ = 0.646, *p* = 0.001, *d* = 1.693, *η*^2^ = 0.417; see Fig. 4).

When controlling for IQ, results from vector levels (SCZ-ASD: *t*_21_ = − 3.961, *p* = 0.001, *d* = 0.845, *η*^2^ = 0.152, SCZ-ADHD: *t*_21_ = -7.771, *p* < 0.0001, *d* = 1.657, *η*^2^ = 0.407, ADHD-ASD: *t*_21_ = − 1.614, *p* = 0.121, *d* = 0.344, *η*^2^ = 0.029) and vector correlations (SCZ-ADHD: *r*_22_ = 0.846, *p* < 0.0001, *d* = 3.173, *η*^2^ = 0.716, SCZ-ASD: *r*_22_ = 0.686, *p* < 0.0001, *d* = 1.885, *η*^2^ = 0.47, ADHD-ASD: *r*_22_ = 0.644, *p* = 0.001, *d* = 1.684, *η*^2^ = 0.415) were confirmed.

When including RSPM correct responses as dependent variable, results from vector levels were confirmed, those from vector correlations replicated the highest correlation between SCZ and ADHD (*r*_23_ = 0.910, *p* < 0.0001, *d* = 4.390, *η*^2^ = 0.828), followed by ADHD and ASD (*r*_23_ = 0.595, *p* = 0.003, *d* = 1.481, *η*^2^ = 0.354), and finally SCZ and ASD (*r*_23_ = 0.552, *p* = 0.006, *d* = 1.324, *η*^2^ = 0.305).

## Discussion

Based on growing evidence of overlapping aetiological and pathophysiological mechanisms between schizophrenia and NDD, we tested to what extent the performance of SCZ, ADHD and ASD overlapped or dissociated, across several ocular-motor tasks and a manual standard RT comparison task, that differed in cognitive processes and loads.

Results highlighted (a) shared inhibition deficits across clinical groups, coherent with our predictions; (b) abnormally high ISV in schizophrenia, unexpectedly as a trend only in ADHD; and (c) prolonged RT across cognitively demanding tasks in schizophrenia. Vector correlations, assessing similarities in the performance impairment between clinical groups, resulted (d) in profiles of deviancy from controls that were highly positively correlated; while vector levels, testing each group’s degree of impairment, indicated (e) the performance of SCZ as the most impaired, followed by ASD and, immediately after, ADHD.

*Ad (a).* Starting with *inhibition*, abnormally frequent MEM premature saccades and ANT direction errors aggregated SCZ, ADHD or ASD, confirming our hypotheses and replicating previous findings^12^.

Deficit in inhibition was the most pervasive in patients with SCZ. SCZ and ASD shared more frequent intrusive saccades towards distractors than TD, confirming the assumption of a dysregulated inhibitory system in both clinical groups. Conversely, the typical intrusive saccade frequency in absence of distractors would suggest intact sustained attention. The same pattern was found in ADHD, disconfirming results from a recent meta-analysis^13^. However, the current study differs from the ones included in the meta-analysis in various ways: participants’ ages (19- versus 8–12-year-olds), task instruction (most studies did not include a distractor condition), task duration (large variability between studies). Likely, one or more of these factors might explain the typical ADHD performance.

Altogether, our data argue in favour of deficient inhibition across ocular-motor and manual-motor systems in schizophrenia, ADHD and, moderately, ASD, suggesting its independence from the motor modality, and possible mediation of inhibition networks (ventro-lateral prefrontal cortex) common in generating both responses^63^.

FMRI studies linked inhibition deficits with disturbances in the fronto-striatal-thalamo-cortical circuits and frontoparietal circuits, previously involved in suppressing reflexive saccades in schizophrenia^64^, ADHD^65^ and ASD^66^. This frontostriatal pathophysiology, that leads to suboptimal inhibition, may underlie the overt symptoms of ADHD.

These findings have various implications. They suggest that inhibition is core to ADHD, as suggested by follow-up behavioural^67^, fMRI^68^ and pharmacological^69^ evidence.

Deficits in inhibition have also been identified early on in the course of ASD^70^ and schizophrenia^71^; they remain stable over time (expressing developmental stability) and have been variously linked to distinct schizophrenia^72^ and ASD^73^ core symptoms, although the nature of such association is less clear than for ADHD symptoms.

Following fMRI evidence, similarities in behavioural inhibition among patients with SCZ, ADHD or ASD could result from overlapping functional abnormalities^63^. Such evidence may suggest overlapping pathophysiologies.

While we present inhibition deficits as a common cognitive abnormality, similar evidence in non-affected first-degree relatives of SCZ and ADHD patients propose inhibition as candidate trans-diagnostic endophenotype and potential genetic vulnerability marker for SCZ and ADHD^72,73^.

*Ad (b)*. Increased *ISV of RT* was found across tasks in SCZ. SDRT and τ best dissociated SCZ from controls with the large effect sizes. Differences in σ from PRO and GNG were associated with medium effect sizes. These results replicate previous ones^37,38^ where increased τ was thought to reflect suboptimal decision processes, while increased σ abnormal sensorimotor processes.

Noteworthy, RT distributions differentiated SCZ from TD across all tasks, proposing ISV as a general measure of cognitive instability as has been suggested already^37^.

Trends of increased ISV in ADHD compared to TD were associated with small effect sizes for SDRT from PRO, ANT and τ from PRO, with medium effect sizes for SDRT from GNG, MEM and τ from GNG. Conversely, σ never dissociated ADHD from TD.

The findings in ADHD could point to a age moderating effect, as suggested elsewhere^39^. Alternatively, they might be adduced to the milder symptom severity of our ADHD group. Replicating previous findings^33,57^, large τ in patients with ADHD and SCZ might suggest this facet of ISV as a trans-diagnostic phenotype of these disorders^57^.

Overall, the decomposition of ISV into several facets contributed to linking different measures of behavioural variability with different and overlapping underlying processes in SCZ^74,76,77^ and ADHD^36,75,78,79^. The present findings do not provide any compelling evidence for atypically high ISV in ASD, coherently with studies showing increased ISV only in those with ASD co-morbid for ADHD^33,42,80^.

Most of the present results remained essentially unchanged when controlling for IQ, with the exception of SDRT in MEM. The determinant of the association of WM with fluid intelligence are still unclear. Carpenter et al.^81^ argued that the increasing difficulty level of the RSPM items was due to the increasing number of required rules to be maintained in WM, appearing as a main mechanism underlying reasoning ability (for alternative explanations, see^82,83^).

More generally, the appropriateness of controlling for IQ has been questioned altogether. Dennis et al.^62^ argued against it, referring to both statistical and theoretical arguments^59^. Overall, while the IQ score in the study of NDD has an undoubtable value, it raises the need for an articulated model that appropriately specifies IQ as latent variable, legitimating the routinary procedure of including it in a model of outcome.

*Ad (c).* Moving to *processing speed*, results replicated findings of deficient volitional responses initiation, but intact processes of simple saccade generation, in SCZ.

Slow responding held for all—manual and ocular-motor—tasks. Furthermore, we found that delayed responses were uncorrelated across saccade and GNG tasks, which may point to distinct response selection deficits with possibly different underlying neuropathological mechanisms in SCZ.

The low correlations with CPE together with the confirmed results when controlling for CPE, may altogether indicate that prolonged response initiation in SCZ is not an effect of daily antipsychotic medication.

ADHD performance was intermediate between SCZ and TD. The descriptive ADHD slowing during ANT (*d* > 0.5) and not during MEM could be explained by the highest cognitive load required in the moment of target appearance in the first task. Typical RT in ASD suggests spared visual orienting^22^.

Conversely, clinical groups shared a typical prosaccade latency, confirming previous studies^23^. Similarly, the typical RT decrease from overlap to gap trials was replicated in all groups alike, suggesting intact neurophysiological processes underlying the gap effect^84^ across patients.

Our findings altogether resulted in *vector levels* depicting SCZ as the group whose performance most strongly dissociated from TD, followed by patients with ASD and then patients with ADHD.

The three profiles of deviancy were positively correlated, as confirmed by the *vector correlations* corresponding to high correlations (0.64 < *r* < 0.89) with large effect sizes, which remained substantially unchanged after removing statistical dependencies between variables, such as those between means and SD, or between dependent variables and IQ, by partialling*.* The fact that large positive correlations resulted from heterogeneous cognitive facets, across distinct psychiatric disorders, suggests that they might reflect a similar profile of deviancy from “normality” for schizophrenia, ADHD and ASD.

Noteworthy, vector correlations evidenced the highest correlation between the profiles of deviancy of SCZ and ADHD, even when including IQ, indicating that these two clinical groups share the most similar profile of impairment.

Conversely, results from vector levels located SCZ closer to ASD along the continuum of impairment, calling for their higher similarity in the severity—rather than the quality—of cognitive impairment.

Back to the neurodevelopmental continuum of impairment^6^, if cognitive deficits are conceptualised as a qualia that manifests across cognitive sub-processes, then this qualia would result in a unique profile of deviancy from “normality” for the three clinical groups, with differences expressing a continuum of cognitive impairment, from schizophrenia as the most to ADHD as the least impaired group.

Current conclusions replicate and strengthen the ones presented in another study from our group^85^ based visual search, confirming the continuum idea with schizophrenia as the group with the most deficient performance and acknowledging the location’s interchangeability of ADHD and ASD along such continuum based on group-specific strengths (e.g., ASD in search).

One might argue that overlapping behavioural outcomes could result from different underlying pathophysiological mechanisms, thus pointing to developmental equifinality (e.g., common endpoint of different developments)^86^. By contrast, the multifinality principle^86^ highlights how similar developmental factors (e.g., overlapping genes) may lead to dissimilar outcomes (e.g., different psychiatric disorders). While we adduce the neurodevelopmental continuum as one possible explanation for overlapping findings among NDD, all cited models are naturally simplifications, likewise they may not be seen as mutually exclusive. Similarly, in the study of schizophrenia, ADHD and ASD, findings of overlapping risk factors parallel evidence of as many unique factors. In this multifactorial conceptualisation of psychiatric disorders the equifinality, multifinality and developmental issues can thus coexist and force to think in terms of complex pathways that lead to a given state^87^.

The present study has some limitations.

First, the sample size of the clinical groups, particularly SCZ, was small, limiting generalisations.

Second, an effect of antipsychotic medications cannot be ultimately excluded. Indeed, it could be argued that prolonged RT resulted from a generalised antipsychotics’ sedative effect. However, low correlation between processing speed and CPE together with unvaried results from ANCOVAs when including CPE as covariate, pointed towards the independency of processing slowing from antipsychotics assumption. Furthermore, for second and third generation antipsychotics the sedative effect is confined to the initial dosage adjustment phase, which did not apply for any of our patients with schizophrenia. Conversely, sedation is not a problem during extended treatment for most patients^88,89^. Ultimately, such argument would also need to explain the typical prosaccade latency in the present SCZ group. Likewise, similar prosaccade speed in patients with schizophrenia before versus after antipsychotic treatment was reported^28,90,91^. Antipsychotic medications seem to also have either no effect or improving effects on antisaccade latency and error rate, opening up to antipsychotics beneficial effects on cognition^92^. Ultimately, it cannot explain the patterns of deviances that SCZ shared with ADHD and ASD.

Third, ANT might be a risk marker for psychosis, rather than a risk for schizophrenia, requiring further investigation.

Fourth, potential effects of fatigue cannot be excluded. However, additional and/or prolonged breaks were provided whenever necessary, while participants who did not complete the battery in one appointment due to tiredness, were invited for a second session.

Fifth, the modest number of trials per task, while being necessary due to the high number of presented tasks and investigated processes, would pose a reliability issue, suggesting the need for replication including more comprehensive data aggregation.

## Conclusion

Overall, these findings emphasize the importance of extending the scope of research from disorder-specific features towards common neurocognitive deficits and their underlying pathophysiologies. Implications for future studies include the need to widen the nosological concept of NDD to include schizophrenia, in order to test the same model across other psychiatric disorders which share genetic liability with NDD and to extend the focus of attention towards intermediate phenotypes common across psychiatric disorders.

## Supplementary Information

Supplementary Information.

## Data Availability

The data from this study are available from the corresponding authors upon request.
