# Supplementary material for: Evidence towards a continuum of impairment across neurodevelopmental disorders from basic ocular-motor tasks
Source: Sci Rep. 2022 Oct 3;12:16521. doi: 10.1038/s41598-022-19661-z (PMC9530118; doi:10.1038/s41598-022-19661-z)
Supplement: Supplementary file 1 — Supplementary Information. [file 41598_2022_19661_MOESM1_ESM.docx]

**Evidence towards a continuum of impairment across Neurodevelopmental disorders from basic ocular-motor tasks**

***Daniela Canu^1^, Chara Ioannou^1^, Katarina Müller^2^, Berthold Martin^2^, Christian Fleischhaker^1^, Monica Biscaldi^1^, André Beauducel^3^, Nikolaos Smyrnis^4,5^, Ludger Tebartz van Elst^6^ and *Christoph Klein^1,4,7^**

^1^Department of Child and Adolescent Psychiatry, Psychotherapy, and Psychosomatics, Medical Centre - University of Freiburg, Faculty of Medicine, University of Freiburg, Freiburg, Germany

^2^Psychotherapeutisches Wohnheim für junge Menschen Leppermühle, Buseck, Germany

^3^Institute of Psychology, University of Bonn, Bonn, Germany

^4^2nd Psychiatry Department, National and Kapodistrian University of Athens, Medical School, University General Hospital "ATTIKON", Athens, Greece

^5^Laboratory of Sensorimotor Control, University Mental Health, Neurosciences and Precision Medicine Research Institute «COSTAS STEFANIS», Athens, Greece

^6^Department of Psychiatry and Psychotherapy, Medical Centre - University of Freiburg, Faculty of Medicine, University of Freiburg, Freiburg, Germany

^7^Department of Child and Adolescent Psychiatry, Medical Faculty, University of Cologne, Cologne, Germany

**a**


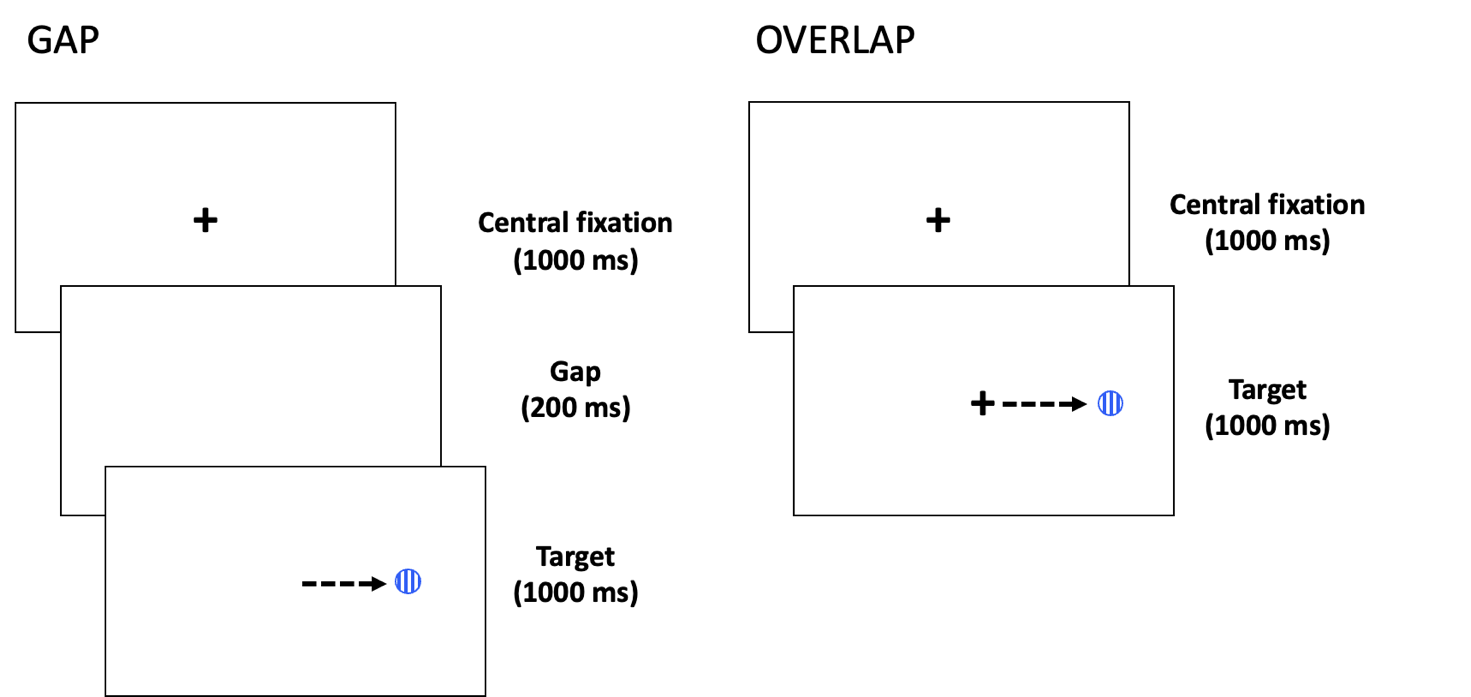


**b**


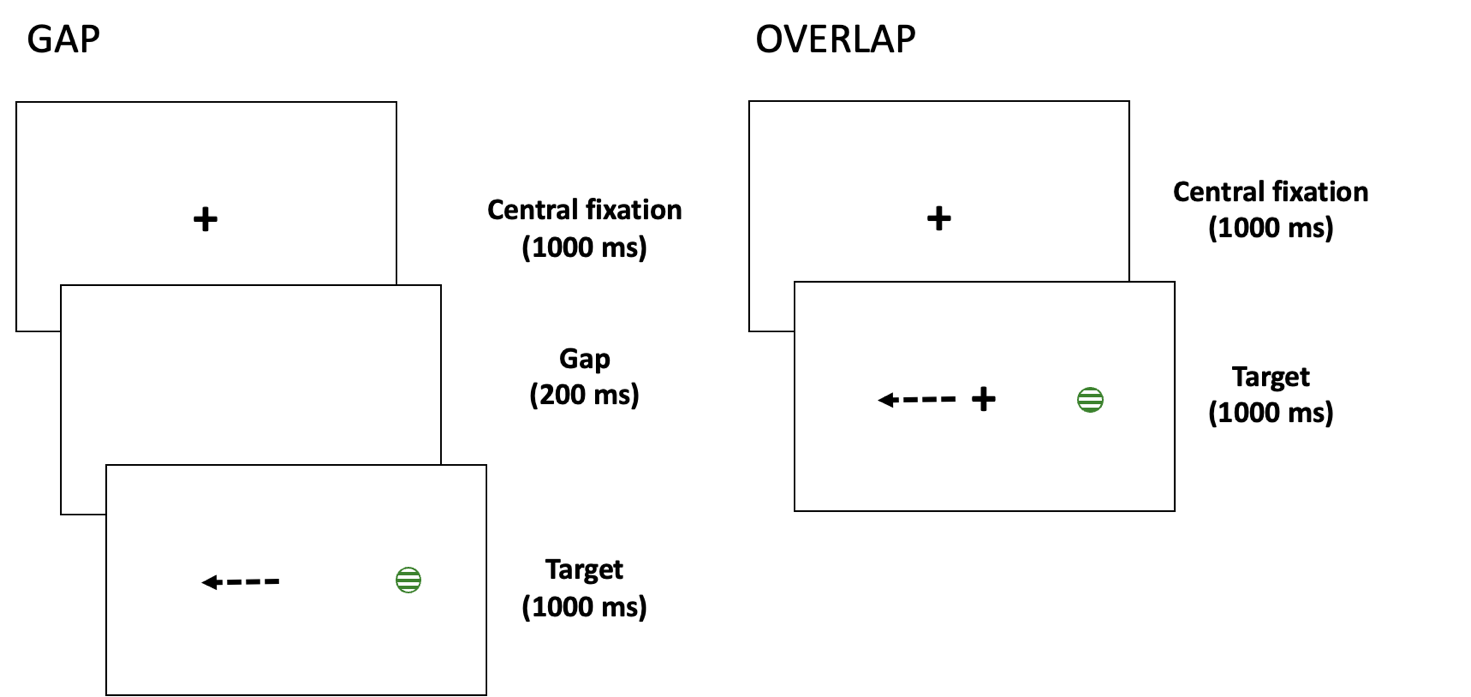


**Supplementary Fig. S1** Representation of the **a** **Gap-Overlap Prosaccade (PRO)** and **b** **Gap-Overlap Antisaccade** **(ANT)** task.

**a –** Subjects fixated a central cross for 1000ms. In the Gap trials a 200ms blank screen preceded the appearance of the target (a coloured, filled dot, subtended visual angle 0.5°), which was presented either 7° left or 7° right of the central cross and extinguished after 1000ms in pseudo-random order without more than three immediate repetitions in the same position. In the Overlap trials, the central fixation remained until the target extinguished (after 1000ms). 100 trials, preceded by eight practice trials, were presented, with constant trial-type alternation (gap, overlap). The inter-trial interval lasted 1000ms.

**b** – This task was identical to the PRO task except that the target was a circle filled with a different colour and subjects were instructed to look straight at its mirror image location in the opposite hemifield.

The arrow indicates the direction of the saccade.


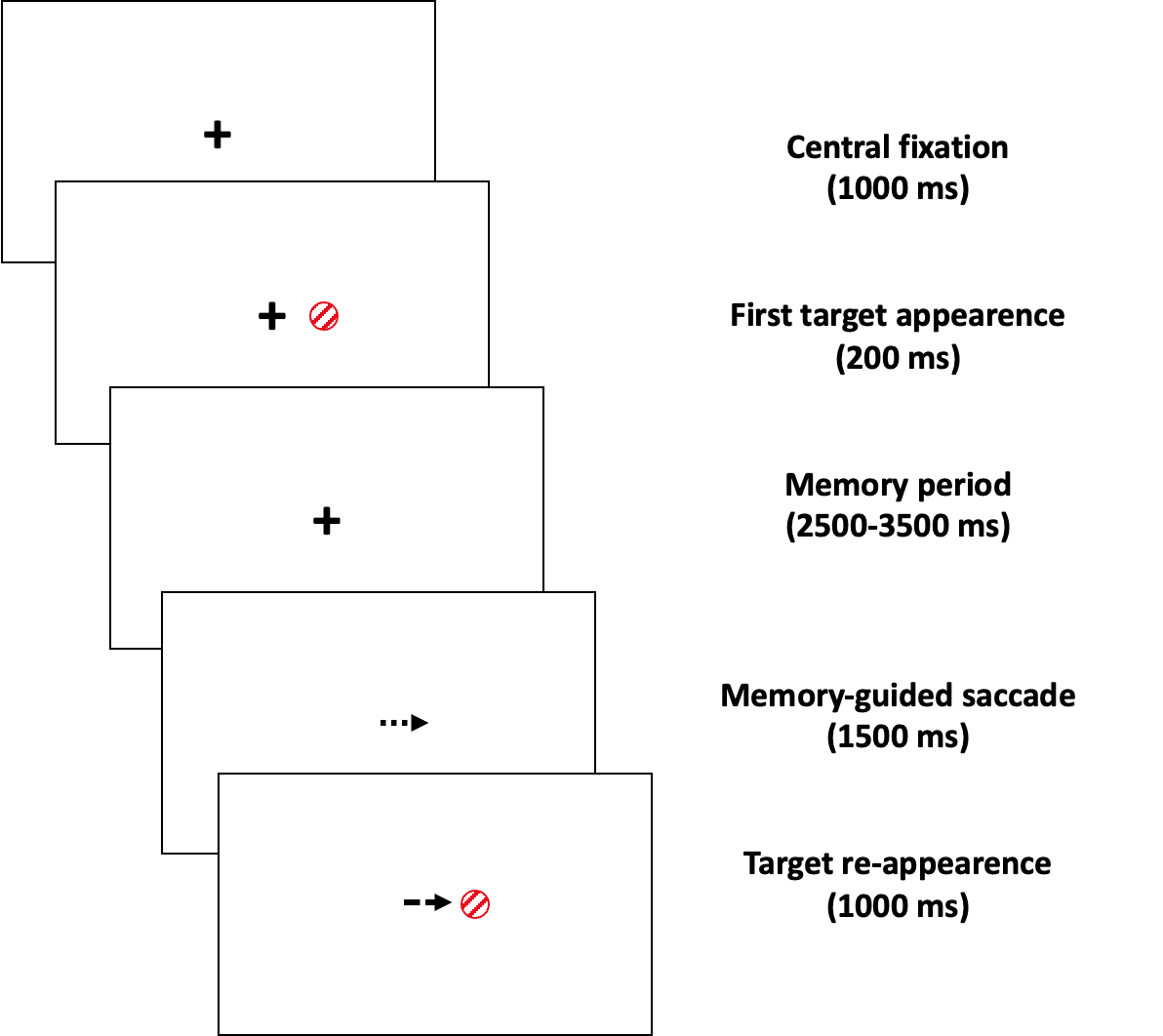


**Supplementary Fig. S2** Representation of the **Memory-guided Saccade** **(MEM)** task.

Each trial began with the presentation of a central fixation cross for 1000ms, followed by a peripheral target (a coloured filled dot, subtended visual angle 0.5°), which appeared for 100ms at one of twelve locations around fixation (±2.5°, ±5°, ±7.5°, ±10°, ±12.5°, ±15°). After a variable delay of 2.5-4.5-sec, the central cross disappeared. Subjects were instructed to produce on cue an eye movement toward the remembered location of a target that had been previously presented in the visual periphery upon the disappearance of the central fixation cross. The task was structured around six practice and 48 experimental trials, whose order was pseudo-randomised based on target locations.

The first arrow indicates the direction of the memory-guided saccade, the second arrow the direction of the visually-guided saccade.

**a**

**b**


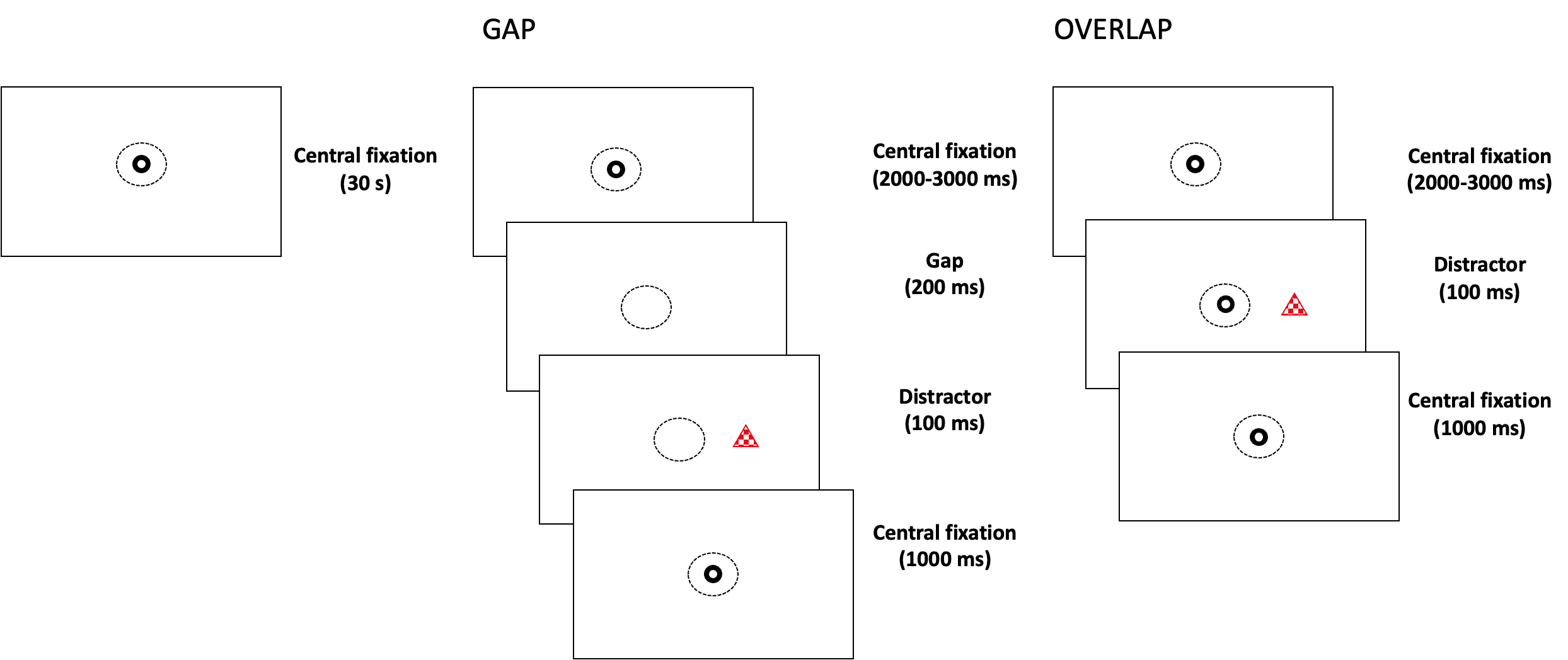


**Supplementary Fig. S3** Representation of the **Fixation (FIX)** task.

The task consisted of five blocks, each of 30-second duration, **a** one block of prolonged fixation without distractors and **b** four blocks of prolonged fixation with distractors. Each block with distractors was built as a sequence of six trials of two trial-types – gap, overlap – constantly alternating, with one distractor presentation per trial. In the gap trials, the central dot remained for a variable 2.0-3.0-sec period, followed by a blank 200ms gap period before the onset of a peripheral distractor, presented at one of four locations (±4°, ±7°) for another 200ms. The reappearance of the central dot for 1000ms preceded the beginning of the following trial. In the overlap trials, the central dot remained while the distractor appeared. The total trial duration was 2,400ms for gap trials and 2,200ms for overlap trials.

The dashed circle indicates within which area participants should fixate.


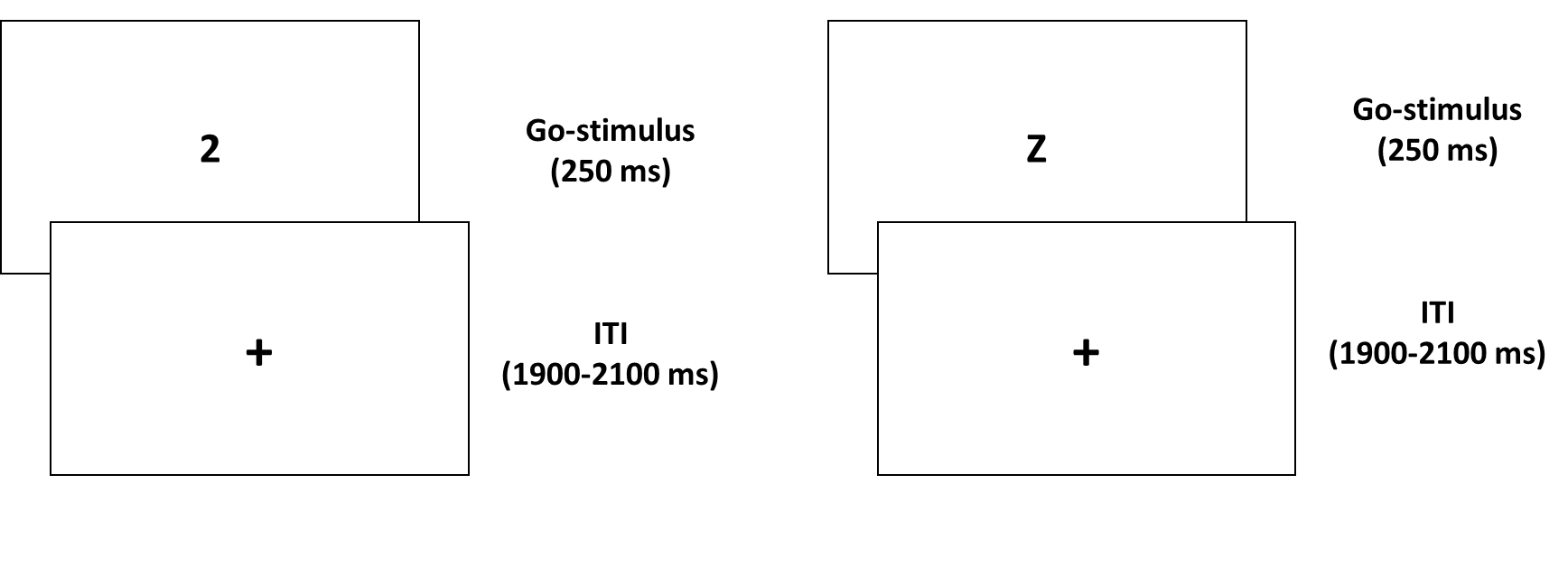


**Supplementary Fig. S4** Representation of **Go/NoGo (GNG)** task.

This task consisted of a pseudo-randomised sequence of alternating Go (number “2”; 86% of trials) and No-Go (letter “Z”; 14% of trials) stimuli, presented in grey colour on a lighter grey-colour background. Ten practice trials were presented, followed by 130 experimental trials. Participants were required to respond as quickly as possible to the Go stimulus (green keyboard button press), but withhold response to the No-Go stimulus. To minimize the discrepancy between decision time and manual reaction time, subjects were monitored to keep their finger on the Go button and maintained their gaze on the screen while responding.

**Supplementary Fig. S5** Percentage of anticipatory saccades from MEM across the six target eccentricities. Error bars represent one standard error of the mean.

The effect sizes of the GROUP*TARGETECCENTRICITY interaction from the repeated-measures ANOVA is also reported here: *η*_p_^2^=0.054

”), MEM anticipatory saccades (“AS_MEM”) and ANT regular direction error (“DE_ANT”). Error bars represent one standard error of the mean. Effect sizes from the between-group subject ANOVA are also reported here.

COM_GNG: *η*_p_^2^=0.098, DE_ANT: *η*_p_^2^=0.139, AS_MEM: *η*_p_^2^=0.148

**Supplementary Fig. S6** Mean response time (RT) of correct response from MEM across the six target eccentricities. Error bars represent one standard error of the mean.

The effect size of the GROUP*TARGETECCENTRICITY interaction from the repeated-measures ANOVA is also reported here: *η*_p_^2^= 0.077

| **Supplementary Table S1.** Post-Hoc Tukey between clinical groups | | | | | | | | | | |
| --- | --- | --- | --- | --- | --- | --- | --- | --- | --- | --- |
| **Task** | **Dependent Variable** | **SCZ - ADHD** | | | **SCZ - ASD** | | | **ADHD - ASD** | | |
|  |  | ***t_99_* ^a^** | ***p*^b^** | ***d*^c^** | ***t_99_* ^a^** | ***p*^b^** | ***d*^c^** | ***t_99_* ^a^** | ***p*^b^** | ***d*^c^** |
| **GNG** | RT | 2.189 | 0.133 | 0.636 | 1.330 | 0.546 | 0.305 | -0.900 | 0.805 | 0.223 |
|  | SDRT | 2.078 | 0.168 | 0.513 | 2.398 | 0.084 | 0.616 | 0.385 | 0.890 | 0.099 |
|  | Mu | 1.167 | 0.649 | 0.303 | -0.218 | 0.996 | 0.049 | -1.493 | 0.446 | 0.424 |
|  | Sigma | 1.818 | 0.271 | 0.403 | 1.697 | 0.331 | 0.426 | -0.101 | 1.000 | 0.031 |
|  | Tau Mean | 1.417 | 0.492 | 0.327 | 2.016 | 0.189 | 0.525 | 0.679 | 0.905 | 0.185 |
|  | Commission errors | 1.051 | 0.720 | 0.286 | 1.538 | 0.419 | 0.461 | 0.550 | 0.946 | 0.146 |
|  | Omission errors | 2.311 | 0.103 | 0.630 | 1.333 | 0.544 | 0.274 | -1.028 | 0.734 | 0.288 |
| **PRO** | CS | -2.713 | 0.039 | 0.762 | -2.319 | 0.101 | 0.535 | 0.384 | 0.981 | 0.105 |
|  | Mean RT | 0.621 | 0.925 | 0.16 | 1.525 | 0.427 | 0.459 | 0.997 | 0.751 | 0.267 |
|  | SDRT | 1.522 | 0.428 | 0.412 | 2.741 | 0.036 | 0.830 | 1.358 | 0.529 | 0.330 |
|  | Mu | -1.099 | 0.691 | 0.282 | -1.631 | 0.366 | 0.437 | -0.600 | 0.932 | 0.160 |
|  | Sigma | 1.794 | 0.282 | 0.470 | 0.941 | 0.783 | 0.260 | -0.901 | 0.805 | 0.275 |
|  | Tau | 1.623 | 0.371 | 0.436 | 3.306 | **0.007** | 1.049 | 1.866 | 0.250 | 0.494 |
|  | AS | 2.404 | 0.083 | 0.645 | 1.518 | 0.431 | 0.342 | -0.926 | 0.791 | 0.264 |
| **ANT** | CS | -3.731 | **0.002** | 0.925 | -4.738 | **<0.0001** | 1.196 | -1.163 | 0.652 | 0.320 |
|  | Mean RT | 2.986 | 0.166 | 0.559 | 2.644 | 0.046 | 0.713 | 0.648 | 0.916 | 0.162 |
|  | SDRT | 1.373 | 0.519 | 0.413 | 1.896 | 0.236 | 0.517 | 0.595 | 0.933 | 0.147 |
|  | AS | 3.392 | **0.005** | 0.987 | 3.482 | **0.004** | 0.895 | 0.156 | 0.999 | 0.038 |
|  | DE | 2.356 | 0.093 | 0.588 | 3.018 | 0.017 | 0.778 | 0.763 | 0.871 | 0.207 |
| **MEM** | CS | -7.333 | **<0.0001** | 1.955 | -8.069 | **<0.0001** | 2.072 | -0.930 | 0.789 | 0.237 |
|  | Mean RT | 0.987 | 0.757 | 0.248 | 1.502 | 0.440 | 0.404 | 0.579 | 0.938 | 0.149 |
|  | SDRT | 0.421 | 0.975 | 0.123 | 1.168 | 0.649 | 0.3 | 0.823 | 0.844 | 0.199 |
|  | AS | 3.392 | **0.005** | 0.987 | 3.482 | **0.004** | 0.895 | 0.156 | 0.056 | 0.038 |
|  | AS_CS | 2.577 | 0.055 | 0.737 | 3.058 | 0.015 | 0.806 | 0.569 | 0.941 | 0.135 |
| **FIX** | Intr. Saccade NO-D | 0.940 | 0.783 | 0.443 | -0.325 | 0.988 | 0.07 | -1.366 | 0.524 | 0.291 |
|  | Intr. Saccade D | 2.405 | 0.083 | 0.698 | 1.392 | 0.887 | 0.33 | -1.066 | 0.711 | 0.260 |
| *Mean RT* mean response time, *SDRT* standard deviation of response time, *CS* correct responses, *AS* anticipatory saccades, *DE* direction errors from the antisaccade task, *AS_CS* correct anticipatory saccades from the memory-saccade task, *Intr. Saccade NO-D* intrusive saccades from block without distractors of the FIX task, *Intr. Saccade* *D* intrusive saccades from block with distractors of the FIX task. ^a^Tuckey’s post-hoc test was used. ^b^Bold typeface = *p* < 0.01. *p* values for all variables indicate significance for differences between pairs of clinical groups. ^c^Cohen’s *d* was used to calculate the effect size. | | | | | | | | | | |

| **Supplementary Table S2.** Correlation between CPE and dependent variables within the SCZ group | | | | |
| --- | --- | --- | --- | --- |
| **Task** | **Dependent Variable** | ***r*^a^** | ***p*^b^** | ***d*^c^** |
| **GNG** | **Mean RT** | **0.307** | **0.188** | **0.645** |
|  | **SDRT** | **0.248** | **0.293** | **0.512** |
|  | Mu | 0.307 | 0.188 | 0.645 |
|  | **Sigma** | **0.274** | **0.256** | **0.570** |
|  | **Tau** | **-0.028** | **0.909** | **0.056** |
|  | **Commission errors** | **-0.133** | **0.635** | **0.268** |
|  | **Omission errors** | **0.295** | **0.207** | **0.618** |
| **PRO** | **CS** | **0.109** | **0.647** | **0.219** |
|  | Mean RT | 0.173 | 0.467 | 0.351 |
|  | **SDRT** | **0.040** | **0.867** | **0.080** |
|  | **Mu** | **0.176** | **0.458** | **0.358** |
|  | **Sigma** | **0.281** | **0.231** | **0.586** |
|  | **Tau** | **0.073** | **0.759** | **0.146** |
|  | **AS** | **0.145** | **0.541** | **0.293** |
| **ANT** | **CS** | **0.133** | **0.577** | **0.268** |
|  | **Mean RT** | **0.092** | **0.698** | **0.185** |
|  | **SDRT** | **0.063** | **0.790** | **0.126** |
|  | **AS** | **0.069** | **0.773** | **0.138** |
|  | **DE** | **-0.123** | **0.606** | **0.248** |
| **MEM** | **CS** | **0.160** | **0.501** | **0.324** |
|  | **Mean RT** | **0.065** | **0.786** | **0.130** |
|  | **SDRT** | **-0.096** | **0.688** | **0.193** |
|  | **AS** | **-0.085** | **0.721** | **0.171** |
|  | **AS_CS** | **-0.076** | **0.751** | **0.152** |
| **FIX** | Intr. Saccade NO-D | 0.039 | 0.870 | 0.078 |
|  | **Intr. Saccade D** | **-0.332** | **0.153** | **0.704** |
| *Mean RT* Mean response time*, SDRT* standard deviation of RT, *CS* correct responses, *AS* anticipatory saccades, *DE* direction errors from the antisaccade task, *AS_CS* correct anticipatory saccades from the memory-saccade task, *Intr. Saccade NO-D* intrusive saccades from block without distractors, *Intr. Saccade* *D* intrusive saccades from block with distractors.  ^a^Pearson correlation coefficient *r* was used. ^b^*p* values for all variables indicate significance for correlation between CPE and dependent variables. ^c^Cohen’s *d* was used to calculate the effect size.  Bold typeface in columns 2-5 highlights the dependent variables on which the SCZ group differed from any other group. | | | | |
